# Experimental Design and Data collection of a finishing end milling operation of AISI 1045 steel

**DOI:** 10.1016/j.dib.2016.01.012

**Published:** 2016-01-15

**Authors:** Luiz Gustavo Dias Lopes, Tarcísio Gonçalves de Brito, Anderson Paulo de Paiva, Rogério Santana Peruchi, Pedro Paulo Balestrassi

**Affiliations:** a FEPI -University Center of Itajubá, Brazil; b UNIFEI-Federal University of Itajubá, Brazil

## Abstract

In this Data in Brief paper, a central composite experimental design was planned to collect the surface roughness of an end milling operation of AISI 1045 steel. The surface roughness values are supposed to suffer some kind of variation due to the action of several factors. The main objective here was to present a multivariate experimental design and data collection including control factors, noise factors, and two correlated responses, capable of achieving a reduced surface roughness with minimal variance. Lopes et al. (2016) [1], for example, explores the influence of noise factors on the process performance.

## **Specifications table**

**Table:** 

Subject area	*Industrial Engineering, Mechanics*
More specific subject area	*Optimization*
Type of data	*Table*
How data was acquired	All the milling experiments were carried out in a FADAL vertical machining center, model VMC 15, with a maximum spindle rotation of 7500 RPM and 15 kW of power in the main motor. The tool overhang was 60 mm. The cutting fluid used in the experiments was synthetic oil Quimatic MEII. The tool used was a positive end mill, code R390-025A25-11M with a 25 mm diameter, an entering angle of *χ*_r_=90°, and a medium step with 3 inserts. Three rectangular inserts were used with edge lengths of 11 mm each, code R390-11T308M-PM GC 1025 (Sandvik-Coromant, 2010). The tool material used was cemented carbide ISO P10 coated with TiCN and TiN via the PVD process. The coating hardness was approximately 3000 HV3 and the substrate hardness 1650 HV3, with a grain size smaller than 1 μm. The work piece material was AISI 1045 steel with a hardness of approximately 180 HB. The work piece dimensions were rectangular blocks with square sections of 100×100 mm and lengths of 300 mm.
Data format	*Row*
Experimental factors	*See*Tables 2*and*3
Experimental features	*Central composite Design of Experiment*
Data source location	*Federal University at Itajubá, Laboratory of Mechanics*
*Data accessibility*	Data is with this article (*See*Table 1)

**Value of the data**

• This unique multivariate experimental design and data collection considers 7 factors that potentially influence the end milling operation of AISI 1045 steel.
• This is a time consuming experimental design with 82 runs. The two surface roughness metrics were measured three times at each of three positions on the work piece, computed after determining the mean of the nine measurements.
• Several optimization techniques could be used to determine the better factor´s levels in this multivariate process.

## 1 Data

The following table presents the central composite experimental design considering 4 control parameters, 3 noise variables and 2 correlated responses. For future research the PC Score was also calculated and incorporated to the table.

## 2 Experimental design, materials and methods

Table 1 shows the central composite experimental design (CCD) combining control factors and noise variables as suggested by [2]. The variables (*x*_1_*, x*_2_*, x*_3_*, x*_4_*, z*_1_*, z*_2_ and *z*_3_) with 10 center points were created. The levels for control and noise factors are described in Tables 2 and 3, respectively.

The different noise conditions express, in some sense, the possible variation that can occur during the end milling operation, such as the tool flank wear (*z*_1_), the variations in cutting fluid concentration (*z*_2_), and the variation of cutting fluid flow rate (*z*_3_). The surface roughness values are expected to suffer some kind of variation due to the action of the combined noise factors.

Measurements of the tool flank wear (*VB*_*max*_) (*z*_1_) were captured with an optical microscope (magnification 45×) with images acquired by a coupled digital camera. The criteria adopted as the end of tool life was a flank wear of approximately *VB*_*max*_=0.30 mm.

The responses measured in the end milling process were *R*_*a*_ (the arithmetic average surface roughness) and *R*_*t*_ (the maximum roughness height-distance from highest peak to lowest valley). These QCs were assessed using a Mitutoyo portable roughness meter, model Surftest SJ 201, with a cut-off length of 0.25 mm. This procedure resulted in 82 experiments, described in Table 3. The two surface roughness metrics were measured three times at each of three positions on the work piece, computed after determining the mean of the nine measurements.

## Figures and Tables

**Table 1:** Experimental Design and Data collection of a finishing end milling operation of AISI 1045 steel.

Run	Control parameters				Noise			Response		*PC Score*^1^
	*x*_1_	*x*_2_	*x*_3_	*x*_4_	*z*_1_	*z*_2_	*z*_3_	*Ra*	*Rt*	*PC*_1_
1	0.10	0.75	300.00	15.00	0.00	5.00	20.00	0.297	2.097	−1.455
2	0.20	0.75	300.00	15.00	0.00	5.00	0.00	1.807	7.587	0.805
3	0.10	1.50	300.00	15.00	0.00	5.00	0.00	0.657	3.467	−0.903
4	0.20	1.50	300.00	15.00	0.00	5.00	20.00	2.573	8.957	1.652
5	0.10	0.75	350.00	15.00	0.00	5.00	0.00	0.353	2.160	−1.400
6	0.20	0.75	350.00	15.00	0.00	5.00	20.00	3.013	9.327	2.050
7	0.10	1.50	350.00	15.00	0.00	5.00	20.00	0.270	1.973	−1.501
8	0.20	1.50	350.00	15.00	0.00	5.00	0.00	2.417	8.743	1.493
9	0.10	0.75	300.00	18.00	0.00	5.00	0.00	0.320	2.087	−1.440
10	0.20	0.75	300.00	18.00	0.00	5.00	20.00	3.170	11.583	2.642
11	0.10	1.50	300.00	18.00	0.00	5.00	20.00	0.280	1.690	−1.553
12	0.20	1.50	300.00	18.00	0.00	5.00	0.00	2.877	10.187	2.133
13	0.10	0.75	350.00	18.00	0.00	5.00	20.00	0.270	2.027	−1.489
14	0.20	0.75	350.00	18.00	0.00	5.00	0.00	3.030	11.197	2.458
15	0.10	1.50	350.00	18.00	0.00	5.00	0.00	0.550	3.340	−1.008
16	0.20	1.50	350.00	18.00	0.00	5.00	20.00	1.520	7.043	0.482
17	0.10	0.75	300.00	15.00	0.30	5.00	0.00	0.497	4.560	−0.788
18	0.20	0.75	300.00	15.00	0.30	5.00	20.00	2.770	10.973	2.222
19	0.10	1.50	300.00	15.00	0.30	5.00	20.00	0.383	2.707	−1.263
20	0.20	1.50	300.00	15.00	0.30	5.00	0.00	3.247	12.473	2.886
21	0.10	0.75	350.00	15.00	0.30	5.00	20.00	0.760	4.647	−0.578
22	0.20	0.75	350.00	15.00	0.30	5.00	0.00	0.800	4.580	−0.563
23	0.10	1.50	350.00	15.00	0.30	5.00	0.00	0.500	3.660	−0.976
24	0.20	1.50	350.00	15.00	0.30	5.00	20.00	2.503	10.757	1.983
25	0.10	0.75	300.00	18.00	0.30	5.00	20.00	0.397	2.877	−1.217
26	0.20	0.75	300.00	18.00	0.30	5.00	0.00	1.063	6.007	−0.070
27	0.10	1.50	300.00	18.00	0.30	5.00	0.00	0.367	2.007	−1.423
28	0.20	1.50	300.00	18.00	0.30	5.00	20.00	2.783	15.330	3.155
29	0.10	0.75	350.00	18.00	0.30	5.00	0.00	0.763	4.217	−0.667
30	0.20	0.75	350.00	18.00	0.30	5.00	20.00	1.437	7.253	0.466
31	0.10	1.50	350.00	18.00	0.30	5.00	20.00	0.383	3.137	−1.172
32	0.20	1.50	350.00	18.00	0.30	5.00	0.00	2.960	11.610	2.495
33	0.10	0.75	300.00	15.00	0.00	15.00	0.00	0.803	4.007	−0.682
34	0.20	0.75	300.00	15.00	0.00	15.00	20.00	2.030	7.213	0.888
35	0.10	1.50	300.00	15.00	0.00	15.00	20.00	0.537	4.583	−0.754
36	0.20	1.50	300.00	15.00	0.00	15.00	0.00	2.110	9.117	1.350
37	0.10	0.75	350.00	15.00	0.00	15.00	20.00	0.920	4.480	−0.497
38	0.20	0.75	350.00	15.00	0.00	15.00	0.00	1.743	7.157	0.668
39	0.10	1.50	350.00	15.00	0.00	15.00	0.00	0.290	2.043	−1.471
40	0.20	1.50	350.00	15.00	0.00	15.00	20.00	0.943	4.460	−0.485
41	0.10	0.75	300.00	18.00	0.00	15.00	20.00	0.513	2.973	−1.112
42	0.20	0.75	300.00	18.00	0.00	15.00	0.00	2.087	7.550	1.001
43	0.10	1.50	300.00	18.00	0.00	15.00	0.00	0.430	2.823	−1.204
44	0.20	1.50	300.00	18.00	0.00	15.00	20.00	2.557	10.570	1.982
45	0.10	0.75	350.00	18.00	0.00	15.00	0.00	0.350	2.457	−1.340
46	0.20	0.75	350.00	18.00	0.00	15.00	20.00	1.700	6.507	0.499
47	0.10	1.50	350.00	18.00	0.00	15.00	20.00	0.617	3.057	−1.019
48	0.20	1.50	350.00	18.00	0.00	15.00	0.00	1.747	8.273	0.907
49	0.10	0.75	300.00	15.00	0.30	15.00	20.00	0.823	4.690	−0.523
50	0.20	0.75	300.00	15.00	0.30	15.00	0.00	3.007	11.787	2.567
51	0.10	1.50	300.00	15.00	0.30	15.00	0.00	0.643	5.230	−0.539
52	0.20	1.50	300.00	15.00	0.30	15.00	20.00	2.937	9.870	2.109
53	0.10	0.75	350.00	15.00	0.30	15.00	0.00	0.803	4.997	−0.473
54	0.20	0.75	350.00	15.00	0.30	15.00	20.00	2.220	9.797	1.574
55	0.10	1.50	350.00	15.00	0.30	15.00	20.00	0.463	2.793	−1.186
56	0.20	1.50	350.00	15.00	0.30	15.00	0.00	2.203	9.823	1.567
57	0.10	0.75	300.00	18.00	0.30	15.00	0.00	0.820	5.343	−0.387
58	0.20	0.75	300.00	18.00	0.30	15.00	20.00	2.547	10.663	1.994
59	0.10	1.50	300.00	18.00	0.30	15.00	20.00	0.377	2.560	−1.299
60	0.20	1.50	300.00	18.00	0.30	15.00	0.00	2.193	8.853	1.354
61	0.10	0.75	350.00	18.00	0.30	15.00	20.00	0.637	4.050	−0.794
62	0.20	0.75	350.00	18.00	0.30	15.00	0.00	2.247	9.590	1.549
63	0.10	1.50	350.00	18.00	0.30	15.00	0.00	0.483	3.400	−1.043
64	0.20	1.50	350.00	18.00	0.30	15.00	20.00	2.887	11.327	2.382
65	0.01	1.13	325.00	16.50	0.15	10.00	10.00	0.100	0.820	−1.868
66	0.29	1.13	325.00	16.50	0.15	10.00	10.00	1	1	1
67	0.15	0.06	325.00	16.50	0.15	10.00	10.00	0.350	1.910	−1.456
68	0.15	2.19	325.00	16.50	0.15	10.00	10.00	1.573	6.817	0.472
69	0.15	1.13	254.29	16.50	0.15	10.00	10.00	0.650	5.257	−0.529
70	0.15	1.13	395.71	16.50	0.15	10.00	10.00	0.440	3.413	−1.072
71	0.15	1.13	325.00	12.26	0.15	10.00	10.00	0.390	3.383	−1.115
72	0.15	1.13	325.00	20.74	0.15	10.00	10.00	1.183	6.230	0.065
73	0.15	1.13	325.00	16.50	0.15	10.00	10.00	0.343	2.990	−1.232
74	0.15	1.13	325.00	16.50	0.15	10.00	10.00	0.540	3.283	−1.027
75	0.15	1.13	325.00	16.50	0.15	10.00	10.00	0.680	4.083	−0.756
76	0.15	1.13	325.00	16.50	0.15	10.00	10.00	0.520	3.247	−1.049
77	0.15	1.13	325.00	16.50	0.15	10.00	10.00	0.540	4.090	−0.856
78	0.15	1.13	325.00	16.50	0.15	10.00	10.00	0.323	2.993	−1.246
79	0.15	1.13	325.00	16.50	0.15	10.00	10.00	0.527	4.990	−0.675
80	0.15	1.13	325.00	16.50	0.15	10.00	10.00	0.607	3.453	−0.942
81	0.15	1.13	325.00	16.50	0.15	10.00	10.00	0.697	4.970	−0.556
82	0.15	1.13	325.00	16.50	0.15	10.00	10.00	0.430	2.863	−1.196

1 The principal component score was computed using Minitab software.

**Table 2:** Control factors and respective levels.

Parameters	Unit	Symbol	Levels				
−2.828	−1.000	0.000	1.000	2.828
Feed rate, *x*_1_	mm/tooth	*fz*	0.01	0.10	0.15	0.20	0.29
Axial depth of cut, *x*_2_	mm	*ap*	0.064	0.750	1.125	1.500	2.186
Cutting speed, *x*_3_	m/min	*Vc*	254	300	325	350	396
Radial depth of cut, *x*_4_	mm	*ae*	12.26	15.00	16.50	18.00	20.74

**Table 3:** Noise factors and respective levels.

Noise factors	Unit	Symbol	Levels		
−1	0	+1
Tool flank wear	Mm	*Z*_1_	0.00	0.15	0.30
Cutting fluid concentration	%	*Z*_2_	5	10	15
Cutting fluid flow rate	l/min	*Z*_3_	0	10	20
